# Fortified breakfast cereal consumed daily for 12 wk leads to a significant improvement in micronutrient intake and micronutrient status in adolescent girls: a randomised controlled trial

**DOI:** 10.1186/s12937-016-0185-6

**Published:** 2016-07-14

**Authors:** Hilary J. Powers, Mark Stephens, Jean Russell, Marilyn H. Hill

**Affiliations:** 1 Human Nutrition Unit, University of Sheffield, Sheffield, UK; 2 Corporate Information and Computing Services, University of Sheffield, Sheffield, UK

**Keywords:** Fortified cereal, Breakfast, Vitamin and mineral status, Micronutrients, Adolescents

## Abstract

**Background:**

Poor micronutrient status is reported among adolescents across Europe and USA. This may be related to the well-documented decline in the regular consumption of breakfast by this group. The regular consumption of a breakfast cereal offers a possible means to improve micronutrient status; fortified cereal is likely to have enhanced benefit. A study was conducted to determine the efficacy of the regular consumption of a fortified cereal with milk, compared with unfortified cereal, consumed either as a breakfast or a supper, in improving micronutrient intake and micronutrient status of adolescent girls.

**Methods:**

A randomised, double-blind, placebo-controlled intervention trial was conducted in girls recruited at ages 16–19 years, from schools and colleges in Sheffield, UK. Girls were randomised to receive 50 g fortified or unfortified cereal, with 150 ml semi-skimmed milk, daily, for 12 weeks, as a breakfast or as a supper. Dietary intake was estimated using a 4-d food diary and blood collected for the assessment of nutritional status. Within-group changes were tested using a paired sample t test; two-way ANOVA was used to analyse effects of the intervention, with cereal type and time of consumption as factors, correcting for baseline values. The analysis was conducted on 71 girls who completed the study.

**Results:**

Consumption of unfortified cereal elicited an increase in the intake of vitamins B_1_, B_2_ and B_6_; consumption of fortified cereal elicited increases in vitamins B_1_, B_2_, B_6_, B_12_, folate and iron (*P* < 0.001) and of vitamin D (*P* = 0.007), all increases were significantly greater than for unfortified cereal. Consumption of the fortified cereal also led to a significant improvement in biomarkers of status for vitamins B_2_, B_12_, folate and of iron, compared with girls receiving the unfortified cereal, and maintained vitamin D status, in contrast with the girls receiving the unfortified cereal (*P* < 0.001).

**Conclusions:**

The daily consumption of cereal with milk for 12 weeks by adolescent girls, increased intakes of micronutrients. The consumption of fortified cereal elicited greater increases than for unfortified cereal and improved biomarkers of micronutrient status. The findings justify strategies to encourage the consumption of fortified cereal with milk by adolescents, either as a breakfast or a supper.

**Trial registration:**

Registered with Current Controlled Trials (Registration: ISRCTN55141306)

## Background

Data from the National Diet and Nutrition Surveys (NDNS) of the UK indicate low intakes of some micronutrients and poor nutritional status among adolescent girls, with concern over vitamin B_2_ (riboflavin), vitamin D, calcium and iron [1]. Dietary data from the National Health and Nutrition Examination Surveys (NHANES) in the USA also show that adolescents are at higher risk of nutritional deficiencies than other age groups in the population [2]_._ Some of these deficiencies may be of particular functional importance to adolescent females given that they are of childbearing age and that low micronutrient intakes in this group influence risk of anaemia, peak bone mass attainment and adverse effects in the offspring [3–7].

Breakfast consumption by children and adolescents has declined over recent decades in many developed countries [8–12]. Milk consumption by children and adolescents in developed countries is also falling, and this is thought to associate with the declining trend in breakfast cereal consumption [1, 13, 14] Studies of association have identified a number of correlates of breakfast consumption in adolescents. Cross sectional studies have reported associations between regular consumption of breakfast and parameters of a healthy lifestyle and some measures of nutritional status [15, 16]. Breakfast-skipping is associated with increased likelihood of being a smoker, of being a consumer of alcohol, of engaging in little exercise and having a higher BMI [17]. On the other hand, some studies report that breakfast consumers have higher energy consumption than non-consumers, and may be associated with an increase in the contribution of fat to total energy intake [18]. A cross–sectional study in Belgium showed that the type of breakfast consumed was associated with the overall dietary intake profile in adolescents [19]. There is some evidence that a breakfast of cereal may have particular value [20] although it is not clear whether associations reported between breakfast cereal consumption and various biomarkers of health are attributable to the consumption of breakfast cereals per se, as part of a daily diet, or the consumption of the cereal as part of a breakfast meal.

There is a definite gender bias in the decline of breakfast consumption, with girls significantly more likely to miss breakfast than boys [17, 20]. There is also an age-related decline in breakfast consumption, with older adolescents more likely to miss breakfast [21]. Reasons often cited are lack of time, loss of appetite in the morning, tiredness or personal choice [22]. Dieting and a desire to lose weight is another reason given for not eating breakfast [23, 24].

The lack of randomised controlled trials to examine hypotheses linking the regular consumption of breakfast cereals to particular health benefits is an obstacle to making specific dietary recommendations.

The study reports that the daily consumption of a fortified cereal with milk, either at breakfast time or supper time for twelve weeks, elicits an increase in micronutrient intake and improvement in biomarkers of micronutrient status in adolescent girls who regularly skip breakfast, that is significantly greater than that observed by the consumption of unfortified cereal with milk. In contrast with girls receiving the unfortified cereal, all girls consuming the fortified cereal achieved the Recommended Nutrient Intake (RNI) for vitamins B_2_, B_6_, B_12_ and folate, although they did not all achieve the RNI for iron, and no girl achieved the current RDA (USA) for vitamin D. The RNI reflects the intake considered to satisfy the requirements of 97.5 % of the age and gender-specific population group.

### Study aim

This study aimed to investigate the efficacy of the regular consumption of fortified cereal with milk, compared with unfortified cereal, either as a breakfast or a supper, in improving micronutrient intake and micronutrient status of adolescent girls who self-report regularly skipping breakfast.

### Hypotheses

We hypothesised that the regular consumption of a fortified breakfast cereal with milk by adolescent girls who often skip breakfast will elicit an increased intake of some micronutrients and improvement in biomarkers of micronutrient status, compared with unfortified cereal. We also hypothesised that the consumption of breakfast cereal at supper-time will elicit the same improvements in nutritional status as when consumed at breakfast time.

## Methods

### Study design

The study was a randomised, placebo-controlled, double-blind intervention trial conducted over a twelve-week period (Sheffield University Breakfast Study – SUBS).

### Recruitment

#### Ethics approval and consent

Ethics approval was obtained from Sheffield University Research Ethics Committee (Ref. SMBRER 223). Informed written consent was taken at the screening visit for the whole study.

### Recruitment

Adolescent girls aged 16–19 years were selected for this study because the National Diet and Nutrition Survey (NDNS) of the UK and data from other developed countries, show that poor nutritional status is prevalent in this group [1, 2]. Girls were recruited from schools, colleges and Universities within the Sheffield area. An Amazon voucher for £30 was offered on completion of the study.

### Eligibility

#### Inclusion criteria

Participants had to be female, aged between 16 and 19 years at the time of recruitment, who self-reported missing breakfast at least 4 times a week. They had to have no diagnosed illness and have low measures of riboflavin status and haemoglobin, as these have previously been found to track well with self-reported breakfast skipping [3].

#### Exclusion criteria

Potential participants were not recruited if they used multivitamin or iron supplements, had donated blood in the previous 6 months, were pregnant or breast feeding or had an allergy to wheat, barley or milk.

### Screening

Recruitment to the study used a website which invited girls who self-reported skipping breakfast at least four times a week. Girls who responded were then screened for low riboflavin status and poor iron status. A screening visit was held to confirm eligibility, to answer any queries and concerns, to take consent, to collect additional information (such as date of birth, address and medication usage) and to take a small finger prick sample (~100 μl) for the measurement of riboflavin and iron status. Consent was taken for both the screening and the main intervention study. Blood samples were analysed for riboflavin status using the erythrocyte glutathione reductase activation coefficient assay (EGRAC) and hemoglobin concentration measured on-site using a portable Hemocue device (Hemocue Hb 201+ DM System, Hemocure, AB, Sweden). EGRAC is the most commonly used biomarker for riboflavin status. Samples with a low riboflavin status have a higher EGRAC value. An EGRAC value of >1.30 is conventionally used to indicate poor riboflavin status [3]. In this study, girls with an EGRAC value >1.40 and a hemoglobin of <137 g/L were eligible for the main study.

### Sample size

Sample size was based on improvement in riboflavin status following the consumption of a fortified cereal with milk. Findings from the RIBOFEM study [3] showed that a supplement of 2 mg riboflavin per day for 8 weeks in young women elicits a fall in EGRAC of 0.25. This cereal study would provide 1.7 mg riboflavin per day for 12 weeks to adolescent girls, in addition to habitual dietary intake. It was anticipated that the intervention would achieve a similar fall in EGRAC and that the baseline SD would be ~ 0.20 on the basis of the RIBOFEM study. Standardised effect size was estimated to be 1.56. It was estimated that we would need 17 girls for each of 4 groups (cereal type and time of intervention) allowing for a possible 25 % dropout in this age and gender group. We therefore aimed to recruit 70 girls to the study.

### Cereal

The cereal, supplied by Kellogg Marketing and Sales Company, was a wheat, rice and barley-based flake similar to their Special K cereal supplied in the UK. Unfortified and fortified cereal was provided and the identity of each was blinded to the researchers and participants. Participants were provided with an equal amount of plain and fruit cereal to add variety and improve compliance. Table 1 shows the estimated macronutrient and micronutrient intakes achieved from the consumption of unfortified and fortified cereal with milk.

**Table 1:** Nutrient and energy supplied by the cereal and milk intervention

Nutrient	50 g fortified cereal	50 g unfortified cereal
	+150 ml milk	+150 ml milk
Energy (kcal)	257	257
Fat (g)	3.1	3.1
Carbohydrate (g)	47.8	47.8
Sugars (g)	15.9	15.9
Vitamin D (μg)	4.15	0.2
Vitamin C (mg)	51.5	1.5
Vitamin B_1_ (mg)	1.21	0.36
Vitamin B_2_ (mg)	1.71	0.61
Niacin (mg)	17.8	1.4
Vitamin B_6_ (mg)	1.74	0.64
Folic Acid (μg)	176	18
Vitamin B_12_ (μg)	1.45	0.60
Iron (mg)	6.5	0.75
Calcium (mg)	215	215

From Windiets and Kellogg’s published data (http://www.kelloggs.co.uk/en GB/special-k-html)

Values are averages of the berry and original varieties

### Intervention and randomisation

Girls who were eligible for the main intervention study were invited to attend three clinics over a period of 12 weeks at the Clinical Research Facility at the Hallamshire Hospital, Sheffield. Prior to attending the first intervention clinic visit, girls were sent a study guide with instructions for the study, a timetable for the scheduled visits and a 4-d food diary to complete. They were also provided with a colour portion guide booklet to facilitate portion size estimation. Girls were instructed to record dietary information for four consecutive days, including one weekend day.

Volunteers were randomised in blocks of twelve to receive a daily intake of either fortified or unfortified cereal (50 g) with semi-skimmed milk (150 ml) for 12 weeks, to be consumed at breakfast time or at supper-time. Volunteers were provided with a graduated measuring jug that indicated the cereal and milk portion sizes. ‘Breakfast’ refers to the first meal of the day, after an overnight fast; ‘supper’ refers to the last food consumed in the day, generally after an earlier complete meal, usually considered as ‘dinner’. Previous experience had indicated that daily consumption of this amount of cereal and milk would be acceptable to volunteers.

#### Visit 1 (week 0)

The purpose of this visit was to collect baseline data and blood samples, to review completed pre-intervention food diaries, and to give the volunteers the first six weeks cereal supply. The girls were weighed using a digital Salter scale and their height measured using a stadiometer for adults. A fasted venous blood sample (6–7 ml) was collected. Girls were given instructions as to how and when to consume the cereal and milk and given tick sheets to record if and when they ate the cereal, for compliance assessment. At this visit, girls were given their next food diary to complete one week prior to visit 2.

#### Visit 2 (week 6)

At this visit the second diet diary was reviewed and girls were given the remainder of the cereal for the study. Any cereal that was not eaten was collected and weighed and used as a second measure of compliance.

#### Visit 3 (week 12)

At this final visit girls provided another venous blood sample and their height and weight were recorded. Any remaining cereal was collected for estimating compliance.

### Blood collection

Blood was collected by venepunture from fasted volunteers in the morning, for plasma biochemical analysis (4.5 ml, EDTA vacutainer) and for routine hematology measurements (2 ml, EDTA vacutainer). Blood cells and plasma were separated by centrifugation, and the plasma divided into aliquots and stored at −80 °C for biochemical analysis. A hemolysate was prepared from the red blood cell pellet and stored at −80 °C for EGRAC measurements [3], to determine vitamin B_2_ status.

### Hematological measurements

Hemoglobin, hematocrit, MCV and red cell number were measured in the hematology laboratory at the Royal Hallamshire Hospital, Sheffield. If the laboratory report indicated ‘suggestive of iron deficiency’ at baseline, on the basis of low hemoglobin, ferritin and MCV, subjects were informed and withdrawn from the study.

### Biochemical techniques

The choice of biochemical measurements of micronutrient status was informed primarily by those nutrients for which there is evidence of poor intake and/or status in adolescent girls, and which might therefore be expected to be influenced by the regular consumption of a fortified cereal.

#### Plasma ferritin for estimating iron stores

Plasma ferritin was measured by ELISA using a commercially available kit (Spectro Ferritin Kit, ATi Atlas Ltd. Chichester, UK). An external quality control was used to monitor accuracy and precision (Randox). The intra-batch coefficient of variation (CV) for QC1 (14 ng/mL) was 3.4 % and for QC2 (80 ng/mL) was 13.1 %. The inter-batch CV for QC1 (14 ng/mL) was 1.6 % and for QC2 (80 ng/mL) was 8.5 %. If the CV for sample replicates was greater than 10 %, these measurements were repeated.

#### Plasma holotranscobalamin (holoTC) for vitamin B_12_ status

Vitamin B_12_ status was assessed as its active form, holotranscobalamin, in plasma, using an ELISA kit (Active-B12 supplied by AXIS-SHIELD). The intra-batch CV for QC1 (25 nmol/L) was 3.1 % and for QC2 (60 nmol/L) was 12.5 %. The inter-batch CV for QC1 (25 nmol/L) was 5.1 % and for QC2 (60 nmol/L) was 3.5 %. If the CV for sample replicates was greater than 15 %, these measurements were repeated.

#### Plasma 5-methyltetrahydrofolate(5-MeTHF) for folate status

Plasma 5-MeTHF was measured by reverse-phase HPLC with fluorescence detection using a method described by Loehrer [25]. 5-MeTHF was detected fluorometrically at excitation and emission wavelengths of 295 nm and 365 nm, respectively. The intra-batch CV for the QC was 2.2 % and the inter-batch CV was 4.6 %.

#### Plasma 25-hydroxyvitamin D (25(OH)D for vitamin D status

The Bone Metabolism Group in the Faculty of Medicine, Dentistry and Health at Sheffield University measured plasma total 25-hydroxyvitamin D using an electrochemiluminescence binding method on a COBAS e 411auto analyser (Roche Diagnostics, Germany). The inter assay CV for total vitamin D was 5.7 %.

#### Erythrocyte glutathione reductase activation coefficient (EGRAC) for vitamin B_2_ status

The activity of erythrocyte glutathione reductase (EC 1.6.4.2) was measured in haemolysates using the method described by Glatzle et al. [26], and modified by Hill et al. [27] for use on the Cobas Autoanalyser, (Roche Diagnostica). Results were expressed as an activation coefficient, EGRAC. The intra batch CV was 2.9 % and the inter batch CV was 1.25 %.

### Dietary assessment

In order to monitor any changes in diet during the intervention and to assess dietary habits in this population of girls, dietary information was collected prior to and during the study. Four-day estimated food intake diaries were used, modified from those developed by the Institute of Food Research (IFR), Norwich, UK. The volunteers were also given food portion booklets developed by IFR and (based on the Ministry of Agriculture, Fisheries and Food (MAFF) food atlas [28] to assist in the recording of portion sizes. Data from the food diaries were analyzed for micronutrient and macronutrient contents with Windiets Research software (version 2010; Robert Gordon University, Aberdeen, United Kingdom).

### Compliance

Two measures were used to assess compliance with the intervention. The completed tick sheets were used to assess self-reported compliance, which is a measure of occasions on which the cereal was consumed. The weight of cereal returned at the end of the study was used to estimate cereal consumed as a percentage of that requested.

### Statistical analysis

Data sets did not deviate significantly from the normal distribution (Kolmogorov-Smirnov test). Data are therefore presented as mean ± SD. Analysis of the effects of the intervention on nutrient and energy intake and biomarkers of nutritional and haematological status, was by two-factor ANOVA (*F* test), with cereal type and time of consumption as factors, correcting for baseline values. When ANOVA indicated a significant difference according to cereal type or time of consumption, a Kruskal-Wallis multiple-comparison z value test was carried out, to identify where the differences lay. Within-group differences between baseline and follow-up were analysed using a paired sample *t* test. Comparison of percentage of girls falling outside normal thresholds for nutritional status was determined using Chi square test. Statistical analysis was performed using SPSS v.20 (IBM, Hampshire, UK). Significance was taken as *P* < 0.05.

## Results

### Subject recruitment, retention and compliance

#### Recruitment

**Fig. 1:**
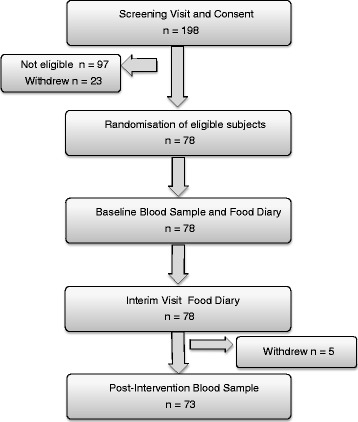
Study protocol

Figure 1 details the flow of participants through the study. 198 girls were screened for moderate riboflavin and iron status, of whom 101 were eligible for the intervention trial. Of these, 78 girls agreed to take part in the main study. Five girls failed to complete the main study (one was withdrawn because of iron deficiency anaemia diagnosed by the Royal Hallamshire Hospital Haematology Department). Data are shown and analyses were conducted on data from 71 girls for whom there was a complete dataset. Of these, 37 girls had been randomised to receive unfortified cereal, 34 to receive fortified cereal. The main study was run alongside the screening and took place over 11 months between July 2012 and May 2013.

### Compliance

On the basis of self-reported occasions on which cereal was consumed, compliance (mean ± SD) to the intervention was 91 ± 7.1 % (*n* = 63 responders). On the basis of the weight of cereal returned, compliance to the cereal consumption was 102 ± 10.3 % (*n* = 71); indicating that some girls consumed more than the weight of cereal they were asked to. Using this metric, compliance was lower in the girls receiving the unfortified cereal (98 ± 10.2 %) than the girls receiving the fortified cereal (105 ± 9.2 %), *P* = 0.004. Girls who consumed their cereal as a ‘breakfast’ had a median time of consumption of 0930 h, the median time of consumption as a ‘supper’ was 2130 h. Time of consumption (as breakfast or supper), did not influence compliance, by either metric.

### Subject characteristics

Table 2 shows subject ages, weight, height and BMI, according to cereal type and time of consumption. The age distribution of girls completing the study was as follows: 5 at 16y, 7 at 17y, 22 at 18y, 36 at 19y and 1 who turned 20y between screening and starting the intervention. Analysis of variance revealed no significant differences in these variables between the randomisation groups.

**Table 2:** Baseline characteristics of girls who completed the intervention

	Unfortified cereal				Fortified cereal				*P* value^e^		
	Morning (*n* = 21)		Evening (*n* = 16)		Morning (*n* = 18)		Evening (*n* = 16)		Cereal type	Timing	Interaction
	Mean	SD	Mean	SD	Mean	SD	Mean	SD			
Height (cm)	165	6.25	165	4.06	164	6.44	164	6.95	0.383	0.853	0.639
Weight (kg)	59.6	5.94	59.5	7.50	62.5	11.35	60.2	10.87	0.397	0.575	0.627
BMI (kg/m^2^)	21.8	2.08	22.0	3.00	23.3	3.34	22.0	2.73	0.178	0.578	0.370
Age (y)	19.0	1.06	19.1	0.70	18.6	1.09	19.1	0.74	0.494	0.225	0.304
Biochemical and hematological status values											
EGRAC^a^	1.71	0.27	1.63	0.22	1.58	0.15	1.59	0.11	0.071	0.519	0.361
Plasma holoTC^b^ (pmol/L)	40.6	16.90	40.08	15.95	34.6	12.68	34.1	14.12	0.143	0.806	0.839
Plasma 5MeTHF^c^ (nmol/L)	14.1	7.74	17.1	8.45	17.0	9.50	17.2	6.18	0.364	0.328	0.557
Plasma 25(OH)D^d^ (nmol/L)	37.1	20.72	42.6	25.63	33.8	21.87	50.3	33.34	0.632	0.062	0.316
Plasma ferritin (ng/ml)	23.9	13.57	18.8	15.41	19.7	11.03	17.9	13.69	0.519	0.360	0.511
Hemoglobin (g/L)	127	7.9	128	8.3	129	6.9	129	9.5	0.235	0.641	0.882
Hematocrit	0.38	0.03	0.38	0.03	0.38	0.02	0.39	0.03	0.967	0.153	0.377
Mean cell volume (fl)	85.8	3.67	84.1	5.01	83.9	3.41	84.4	4.49	0.348	0.451	0.348

^a^erythrocyte glutathione reductase activation coefficient

^b^holotranscobalamin

^c^5-methyltetrahydrofolate

^d^25-hydroxyvitamin D

^e^differences between groups according to cereal type, timing of consumption, and interactions

### Baseline biochemistry

#### Markers of micronutrient and hematological status

Table 2 also shows values for biochemical and hematological variables at baseline, according to cereal type and time of consumption. Analysis of variance revealed no differences in any of these variables between randomisation groups at baseline. Mean (±SD) EGRAC was higher (1.63 ± 0.21 vs 1.52 ± 0.20) and mean plasma ferritin (20 ± 13.4 vs 28 ± 22.9 μg/L) and hemoglobin concentrations were lower (128 ± 8.1 vs 131 ± 8.78 g/L) than the means (±SD) for girls of this age group in the UK, reported in NDNS [1]. Mean (±SD) plasma 25(OH)D concentration (40.8 ± 25.9) was comparable with the NDNS mean (±SD) of 42.5 ± 21.5 nmol/L.

### Adequacy of micronutrient status

All girls fell above the conventional threshold of >1.30 for EGRAC, indicating low riboflavin status. 46 % the girls had evidence of low vitamin B_12_ status (plasma holoTC < 37 pmol/L and iron status (plasma ferritin < 15 ng/mL) whilst 32 % of the group had low vitamin D status (plasma 25(OH)D < 25 nmol/L).

### Dietary intakes

Intakes of macronutrients and energy at baseline are shown in Table 3. There were no differences in any variable between cereal groups or according to time of consumption but there was an interaction between these factors for non-milk extrinsic sugars (NMES) sugars (g), sugars (% energy), total carbohydrate (g) and carbohydrate (% energy).

**Table 3:** Daily intakes of macronutrients and energy at baseline^a^

	Unfortified cereal				Fortified cereal				*P* value^d^		
	Morning (*n* = 21)		Evening (*n* = 16)		Morning (*n* = 18)		Evening (*n* = 16)		Cereal type	Timing	Interaction
	Mean	SD	Mean	SD	Mean	SD	Mean	SD			
Energy (kJ)	6756	2203	7318	2196	7712	2144	6426	2108	0.921	0.521	0.092
Fat (g)	61	26.2	65	25.6	71	25.0	61	24.6	0.644	0.696	0.253
Fat (% energy)	33.6	6.33	32.5	6.32	32.9	6.16	34.1	6.04	0.793	0.975	0.453
Protein (g)	59	19.8	57	10.0	63	19.2	58	18.9	0.567	0.471	0.809
Carbohydrate (g)	196	67.3	229	67.3	238	65.3	187	64.3	0.963	0.582	0.011
Carbohydrate (% energy)	46.3	6.24	50.8	6.00	49.9	71.28	46.8	6.85	0.870	0.693	0.010
Sugars (g)	72	37.3	96	36.7	101	36.3	70	36.0	0.814	0.749	0.003
Sugars (% energy)	16.7	6.02	20.1	5.96	21.9	5.79	17.0	5.69	0.394	0.662	0.006
Starch (g)	113	42.9	122	4.2	126	41.7	104	41.0	0.806	0.452	0.114
NMES^b^ (g)	50	33.5	75	33.0	75	32.6	48	27.6	0.947	0.945	0.002
NSP^c^ (g)	8.9	3.72	9.9	3.71	11.1	3.68	9.7	3.64	0.251	0.813	0.184
Alcohol (g)	11.9	12.22	8.6	12.0	6.33	11.9	6.0	11.69	0.190	0.574	0.557
Alcohol (% energy)	5.1	5.20	2.5	5.47	2.89	5.26	3.14	5.65	0.403	0.592	0.231

^a^energy and nutrient intakes determined using 4-day estimated diet diaries, aided by the use of portion size booklets

^b^non-milk extrinsic sugars

^c^non-starch polysaccharides

^d^differences according to cereal type, time of consumption and interaction

Intakes of micronutrients are shown in Table 4. There were no differences in intakes between cereal groups or according to time of consumption but there was an interaction between these factors for folate intake. At least half of the girls had intakes of vitamin B_2_ and B_6_, folate, iron and calcium lower than both the UK Recommended Nutrient Intakes (RNIs) and the Dietary Reference Intakes (DRIs) for USA. There are currently no DRV’s for vitamin D for people between the ages of 4 and 65y in the UK [29], and therefore comparison was made with the recommendation from the Institute of Medicine (IOM) [30]. Intakes of vitamin D were lower than the IOM Estimated Average Requirement (EAR) of 10 μg per day in all of the girls.

**Table 4:** Daily intakes of micronutrients at baseline^a^

	Unfortified cereal				Fortified cereal				*P* value^b^		
	Morning (*n* = 21)		Evening (*n* = 16)		Morning (*n* = 18)		Evening (*n* = 16)		Cereal type	Timing	Interaction
	Mean	SD	Mean	SD	Mean	SD	Mean	SD			
Vitamin B_1_ (mg)	1.08	0.46	1.09	0.44	1.33	0.46	0.98	0.44	0.490	0.109	0.095
Vitamin B_2_ (mg)	0.97	0.41	1.06	0.40	1.28	0.42	0.98	0.4	0.200	0.366	0.068
Niacin (mg)	24.2	9.71	25.0	9.68	28.3	9.58	26.2	9.40	0.228	0.830	0.576
Vitamin B_6_ (mg)	1.17	0.51	1.27	0.48	1.50	0.46	1.21	0.48	0.321	0.334	0.065
Vitamin B_12_ (μg)	3.32	1.66	2.76	1.64	2.83	1.64	2.70	1.60	0.541	0.438	0.539
Folate (μg)	138	61	161	60	189	60	148	59	0.176	0.606	0.039
Vitamin D (μg)	1.55	1.33	1.51	1.32	1.93	1.30	1.53	1.28	0.501	0.525	0.620
Calcium (mg)	676	283	677	282	771	280	631	274	0.637	0.360	0.358
Sodium (mg)	2373	805	2480	804	2449	794	2167	776	0.609	0.725	0.365
Iron (mg)	8.06	2.57	8.51	2.60	9.54	2.56	7.45	2.52	0.699	0.207	0.050

^a^nutrient intakes determined using 4-day estimated diet diaries, aided by the use of portion size booklets

^b^differences according to cereal type, time of consumption, and interaction

### Effects of the intervention

#### Effects of the intervention on nutritional status and haematological biomarkers

The effects of the intervention on biochemical and hematological variables are shown in Table 5. Data are presented for values before and after intervention, according to cereal type. Two-way ANOVA revealed no effect of the time of consumption of the cereal on any variable and therefore the table shows results for morning and evening consumption combined, for the fortified and unfortified cereal.

**Table 5:** Nutritional status and hematological biomarkers following intervention

Analyte	Unfortified cereal (*n* = 37)		Fortified cereal (*n* = 34)		*P* value^a^
	Pre-intervention	Post-intervention	Pre-intervention	Post-intervention	
	Mean (SD)	Mean (SD)	Mean (SD)	Mean (SD)	
EGRAC^b^	1.68 (0.25)	1.65 (0.18)	1.58 (0.13)	1.38 (0.15)*	<0.001
Plasma holoTC^c^ (pmol/L)	40.7 (16.27)	39.4 (7.44)	35.3 (12.94)	42.8 (13.66)**	<0.001
Plasma 5MeTHF^d^ (nmol/L)	15.4 (8.09)	14.1 (6.90)	17.3 (7.96)	32.9 (16.16)**	<0.001
Plasma 25(OH)D^e^ (nmol/L)	39.4 (22.77)	30.6 (18.74)*	42.5 (29.21)	43.7 (28.22)	<0.001
Plasma ferritin (ng/ml)	21.7 (14.42)	18.4 (11.63)	19.3 (12.38)	22.1 (16.73)**	<0.001
Hemoglobin (g/L)	128 (8.03)	128 (6.63)	130 (7.96)	130 (7.68)	0.876
Hematocrit	0.38 (0.03)	0.38 (0.02)	0.38 (0.02)	0.38 (0.02)	0.687
Mean cell volume (fl)	85.0 (4.31)	84.1 (4.33)*	84.0 (3.84)	83.2 (3.82)*	0.876

*significantly lower than value pre-intervention (paired *t* test; *P* < 0.05)

**significantly higher than value pre-intervention (paired *t* test; *P* < 0.05)

^a^significance of difference between cereal groups after intervention, corrected for baseline (ANOVA)

^b^erythrocyte glutathione reductase activation coefficient

^c^holotranscobalamin

^d^5-methyltetrahydrofolate

^e^25-hydroxyvitamin D

Paired sample *t* test revealed a significant fall (improvement) in EGRAC, and a significant increase in plasma holotranscobalamin, plasma 5-MeTHF and plasma ferritin after the intervention in the girls receiving the fortified cereal. Plasma 25(OH)D concentration fell in the girls receiving the unfortified cereal, but was maintained at the pre-intervention value in the group receiving the fortified cereal. Mean cell volume fell in both groups. When the effect of the intervention was compared between cereal types, girls eating the fortified cereal showed a significant improvement in biomarkers of vitamin B_2_, vitamin B_12_, folate, and vitamin D status and of plasma ferritin, compared with girls receiving the unfortified cereal.

#### Effects of intervention on the percentage of girls with nutrient biomarkers below the lower limits for normality

Table 6 shows the percentage of girls who would be considered as ‘deficient’ in specific micronutrients, according to the stated threshold for each biomarker. Data are shown as percentages before and after the intervention, according to cereal type. Three thresholds are used for EGRAC; the conventional threshold is 1.30 but it has been suggested that a higher threshold should be used to reflect a functional deficiency [3], therefore 1.40 and 1.60 are used too. For girls receiving the fortified cereal, there was a significant decrease in the percentage with biomarker values below the lower limit of normality (or above the EGRAC thresholds) when compared with girls receiving unfortified cereal, for vitamin B_2_, vitamin B_12_ and folate.

**Table 6:** Effects of cereal intervention on percentage of girls falling below^a^ biochemical thresholds for normality

Analyte	Threshold	Unfortified cereal		Fortified cereal	
		Pre-intervention	Post-intervention	Pre-intervention	Post-intervention
EGRAC^b^	>1.3	100	100	100	63*
EGRAC	>1.4	95	97	94	42*
EGRAC	>1.6	60	57	47	8*
Plasma HoloTC^c^	<37pmol/L	46	51	53	36*
Plasma 5MeTHF^d^	<14 nmol/L	11	8	14	0*
Plasma 25(OH)D^e^	<25 nmol/L	32	46	31	31
Plasma ferritin	<15 ng/ml	46	49	50	44
Hemoglobin	<120 g/L	24	14	11	15
Mean cell volume (fl)	<84 fl	27	49	46	53

*significantly different from girls in the unfortified group (Chi Squared test; *P* < 0.05)

^a^above threshold values for EGRAC

^b^erythrocyte glutathione reductase activation coefficient

^c^plasma holotranscobalamin

^d^plasma 5-methyl tetrahydrofolate

^e^plasma 25-hydroxyvitamin D

### Effects of intervention on nutrient intakes

#### Macronutrients and energy

There were no significant differences in post-intervention values between consumers of unfortified and fortified cereal. Within-group analysis revealed a significant decrease from the pre-intervention value for fat and carbohydrate as a percentage of energy, and an increase in starch (Table 7). An increase in protein and carbohydrate was observed which reached statistical significance for the consumers of unfortified and fortified cereal, respectively. Alcohol intake as a percentage of energy showed a decrease from baseline in response to cereal intervention; there was no effect of cereal type.

**Table 7:** Intakes of macronutrients and energy following intervention^a^

	Unfortified cereal (*n* = 37)		Fortified cereal (*n* = 34)		
Nutrient	Pre-intervention	Post-intervention	Pre-intervention	Post-intervention	*P*-value^d^
	Mean (SD)	Mean (SD)	Mean (SD)	Mean (SD)	
Energy (kcals)	1665 (526)	1732 (377)	1695 (534)	1743 (329)	0.952
Fat (g)	63 (23)	62 (18)	67 (29)	58 (19)	0.269
Fat (% energy)	33 (6)	31 (5)*	33 (7)	29 (6)*	0.078
Protein (g)	59 (19)	70 (16)**	61 (20)	67 (17)	0.357
Carbohydrate (g)	210 (66)	230 (53)	214 (74)	242 (45)**	0.351
Carbohydrate (% energy)	48 (6)	51 (6)**	48 (7)	53 (6)**	0.063
Sugars (g)	82 (41)	81 (32)	87 (37)	92 (32)	0.173
Sugars (% energy)	18 (6)	18 (5)	20 (7)	20 (6)	0.086
NMES^b^ (g)	61 (37)	55 (27)	63 (34)	63 (32)	0.273
Starch (g)	116 (32)	142 (32)**	115 (53)	137 (30)**	0.441
NSP^c^ (g)	9 (3)	10 (3)	10 (4)	10 (3)	0.734
Alcohol (g)	11 (15)	4 (7)*	6 (8)	5 (8)	0.304
Alcohol (% energy)	4 (6)	2 (3)*	3 (4)	2 (3)*	0.359

*significantly lower than value pre-intervention (paired *t* test; *P* < 0.05)

**significantly higher than value pre-intervention (paired *t* test; *P* < 0.05)

^a^energy and nutrient intakes determined using 4-day estimated diet diaries, aided by the use of portion size booklets

^b^non-milk extrinsic sugars

^c^Non-starch polysaccharides

^d^significance of differences between cereal groups, corrected for baseline

#### Vitamins, minerals and trace elements

Within-group analysis showed that consuming the unfortified cereal improved intake of vitamins B_1,_ B_2_ and B_6,_ and consuming the fortified cereal improved intakes of all fortificants - vitamins B_1_, B_2_, niacin, B_6_, B_12_, C, D, folate and iron (Table 8). The intake of these nutrients was significantly greater than for girls receiving unfortified cereal; there was no effect of time of consumption. There was no difference between groups in the effect of the interventions on calcium intakes, although within-group analysis showed an increase in the girls receiving the milk with unfortified cereal.

**Table 8:** Daily intake of micronutrients following intervention^a^

Nutrient	Unfortified cereal (*n* = 37)		Fortified cereal (*n* = 34)		*P* value^b^
	Pre-intervention	Post-intervention	Pre-intervention	Post-intervention	
	Mean (SD)	Mean (SD)	Mean (SD)	Mean (SD)	
Vitamin B_1_ (mg)	1.1 (0.40)	1.3 (0.35)**	1.2 (0.50)	2.2 (0.36)**	<0.001
Vitamin B_2_ (mg)	1.0 (0.41)	1.4 (0.35)**	1.2 (0.43)	2.7 (0.50)**	<0.001
Niacin (mg)	25 (8.1)	27 (9.0)	27 (11.1)	41 (10.3)**	<0.001
Vitamin B_6_ (mg)	1.2 (0.45)	1.8 (0.40)**	1.3 (0.53)	3.0 (0.64)**	<0.001
Vitamin B_12_ (μg)	3.1 (1.81)	2.8 (1.09)	2.8 (1.44)	4.1 (1.78)**	<0.001
Folate (μg)	148 (55)	151 (61)	171 (69)	314 (54)**	<0.001
Vitamin D (μg)	1.5 (1.35)	1.6 (0.91)	1.8 (1.28)	5.5 (1.32)**	0.007
Calcium (mg)	677 (282)	822 (248)**	713 (282)	775 (177)	<0.001
Sodium (mg)	2419 (7367)	2528 (631)	2335 (863)	2576 (637)	0.628
Iron (mg)	8.3 (2.47)	7.7 (2.37)	8.9 (2.86)	13.1 (2.31)**	<0.001

**significantly higher than pre-intervention (paired *t* test; *P* < 0.05)

^a^nutrient intakes determined using 4-day estimated diet diaries, aided by the use of portion size booklets

^b^significance of difference between cereal groups corrected for baseline values (ANOVA)

#### Effect of intervention on percentage of girls with intakes below recommended intakes

Intakes of micronutrients following the intervention were compared with RNIs for the UK and DRIs for USA. After intervention with fortified cereal all girls had intakes of vitamins B_2_ and B_6_ that exceeded both sets of recommendations. Intakes of folate and vitamin B_12_ also achieved UK recommendations but fell short of DRIs; no girl achieved the recommended DRI for folate (Table 9). A high percentage of girls in this group still had iron intakes below both sets of recommendations, but none of the girls had an iron intake less than the Lower Reference Nutrient Intake (LRNI) for the UK, which represents an intake that satisfies the requirement of only 2.5 % of the population. In contrast, after intervention with unfortified cereal, micronutrient intakes less than both sets of recommendations persisted across all micronutrients with the exception of vitamin B_6_. Although calcium was not a fortificant, the cereal and milk intervention increased calcium intakes but overall, intakes fell short of RNIs and DRIs in at least 50 % of girls, after the intervention. Although vitamin D intakes increased significantly in the fortified group, and were higher in the fortified group post-intervention, than the unfortified group, intakes still fell short of the EAR of 10 μg proposed by IOM [30].

**Table 9:** Effect of intervention on percentage of girls with micronutrient intakes below recommendations

Diet	% below RNI Unfortified cereal		% below RNI Fortified cereal		% below DRI/RDA^a^ Unfortified cereal		% below DRI/RDA^a^ Fortified cereal	
	Pre	Post	Pre	Post	Pre	Post	Pre	Post
Vitamin B-2	62.2	18.9	50	0	62.2	18.9	50	0
Vitamin B-6	54.1	9.5	50	0	54.1	9.5	50	0
Vitamin B-12	18.9	13.5	25	0	35.1	35.1	44.1	14.7
Folate	78.4	86.5	72.2	0	100	100	100	100
Iron	97.3	100	97.2	82.9	97.3	100	97.2	82.9
Calcium	70.3	48.6	66.7	60	97.3	94.6	97.1	97.1
Vitamin D^a^	N/A	N/A	N/A	N/A	100	100	100	100

pre, post refer to values before and after intervention, *RNI* recommended nutrient intake (UK), *DRI* Dietary Reference Intake (USA) ^a^RDA (Recommended Dietary Allowance) from the Institute of Medicine Report (31)

Two-way ANOVA, correcting for baseline showed no effect of cereal type or time of consumption on weight or BMI. However, analysis of weight and BMI for all evening consumers, regardless of whether the cereal was fortified or not, showed a small but significant increase in weight (mean ± SD, 0.93 ± 1.96 kg) over the intervention period (*P* = 0.012), which was not seen in girls consuming the cereal in the morning (mean ± SD, 0.27 ± 1.64 kg).

## Discussion

The consumption of fortified cereal with milk elicited very clear benefits in terms of increased intakes of some vitamins and iron, and significant improvements in biomarkers of nutritional status. The beneficial effects were seen whether the cereal with milk was consumed in the morning or the evening. The consumption of unfortified cereal with milk also showed some benefit but this was restricted to a few nutrients and was modest compared with the fortified cereal. This observation is important because previous statements about the health benefits of breakfast consumption in any age group have relied almost entirely on observational studies, which could only show associations. Girls taking part in the study had micronutrient intake and status profiles comparable with those reported in the NDNS, so the findings can be considered relevant to their gender and age group.

After 12 weeks of consuming a fortified cereal with milk each day all girls achieved intakes of at least RNI for all micronutrients except for iron and calcium. Intakes were still lower than the DRI for folate and vitamin D for all the girls. Several observational studies have shown that regular breakfast consumption among young people is associated with higher intakes of some micronutrients, including folate, vitamin D, calcium, iron and zinc [31, 32], although details of the type of breakfast consumed are often lacking. Concern has been expressed about a low intake of vitamin D in some sections of the UK population, and there is evidence for poor vitamin D status in some groups [1]; low intakes of this vitamin in adolescents are reported for several other European countries [33]. Vitamin D intake increased significantly in the girls receiving the fortified cereal and this was sufficient to maintain vitamin D status over the period of the intervention, in contrast with the girls receiving the unfortified cereal, in whom vitamin D status fell during the study. This is important because there are few good dietary sources of vitamin D [34] and people living in the Northern hemisphere generally show a decline in plasma concentrations of 25(OH)D during the winter months [35]. The study was conducted during winter and summer months, when potential for dermal synthesis of vitamin D would have been different but the block randomisation method would have minimised differences in any seasonal effect on vitamin D synthesis between the groups. The regular consumption of a cereal fortified with vitamin D could make a useful contribution to dietary vitamin D and evidently has the potential to mitigate the seasonal deterioration in vitamin D status.

This simple intervention had a clear beneficial effect in terms of nutrient intake in this vulnerable group. This was true whether the cereal was consumed in the morning or the evening, offering potentially important flexibility in terms of a public health message.

There is very limited evidence that the consumption of a fortified breakfast cereal leads to an improvement in micronutrient status, due to the lack of randomised controlled trials. Tucker and colleagues [36] conducted a study in adults aged 50-85y, focussed on effects of fortified cereal on plasma homocysteine concentration. They reported improvements in vitamin B_6_, B_12_ and folate status as well as a decrease in homocysteine concentration. Whilst this is of interest, adolescents are more likely to skip breakfast [16, 21, 22], making it particularly important to demonstrate beneficial effects of breakfast cereal consumption in this group. In our trial, compared with the girls receiving unfortified cereal, consumption of the fortified cereal led to significant improvements in biomarkers of vitamin B_2_, B_12_, vitamin D and folate status as well as plasma ferritin, a biomarker of hepatic iron stores. Despite the increase in iron stores elicited by the fortified cereal intervention compared with the unfortified cereal, no increase in hemoglobin was observed and there was no difference in hemoglobin concentration between the two groups following the intervention. An increase in iron stores following the intervention would suggest that iron delivery to the site of hemoglobin synthesis was adequate, and that hemoglobin synthesis was not compromised at the outset. Additional available iron in the diet would then be stored as hepatic iron. There were no differences in mean hemoglobin at baseline between the cereal groups but fewer girls receiving fortified cereal had low hemoglobin (<12 g/L), which may have influenced ferritin responses in this group. An intervention study carried out in New Zealand among women with low iron stores, demonstrated a beneficial effect on iron status of consuming an iron-fortified breakfast cereal together with kiwi fruit, which has a high vitamin C content, compared with cereal consumed with banana [37]. Although a direct comparison of this study with the present study is difficult to make, given differences in age of the women, and iron status at the outset, the results suggest that the vitamin C content of fortified cereal may be inadequate to maximise absorption of cereal iron.

There is very little information available regarding the nutritional status of adolescent breakfast consumers compared with non-consumers. The majority of studies have paid more attention to obesity and measures of metabolic health than biomarkers of nutritional status. A recent study of younger children in Cyprus reported no difference in plasma ferritin concentration between consumers and non-consumers [38].

Despite the unequivocal improvement in micronutrient status in the girls receiving fortified cereal, and the decrease in the percentage of girls falling below the biomarker thresholds for normality, the improvement was insufficient to bring all girls into the normal range for conventional biomarkers of status except for folate. The normalisation of folate status is of particular relevance to this study population, given the importance of good folate status to healthy pregnancy [39].

Girls consuming cereal in the evening showed a small but significant increase in weight, which was not seen in the girls consuming cereal in the morning. The two-way ANOVA, correcting for baseline values, did not reveal any difference between groups, suggesting that undue emphasis should not be placed on the within-group finding. It is possible that cereal consumed in the evening is less likely to displace other foods, but this was not reflected in a significant increase in energy intake in the evening consumers. Others have explored effects of breakfast habits on energy intake and there have been several reports that breakfast skipping causes a compensatory increase in energy intake later in the day [40]. There may be differences in physical activity following cereal consumed as a breakfast or as a supper. Betts et al. [41] recently reported that daily breakfast consumption was associated with an increase in energy expenditure during the morning of the breakfast. The mean weight gain reported in our study was small and it will be important to know whether this was maintained or lost if evening cereal consumption was sustained over a longer period.

The cereal and milk intervention led to a modest but significant decrease in the proportion of dietary fat as a percentage of total energy intake and an increase in the proportion of energy derived from carbohydrate. This, together with the lack of increase in energy intake suggests that the cereal may have displaced fat-rich foods from the diet of these girls. This is compatible with the findings of Deshmuk-Taskar et al. [42], who reported a lower intake of fat and a higher intake of carbohydrate in children and adolescents who consumed ready to eat breakfast cereal (RTEC) compared with non-consumers. There is also a small literature suggesting that breakfast consumption may influence snacking behaviour such as to reduce energy intake from snacks and reduce snacking frequency [43]. Utter et al. [44] reported that skipping breakfast is associated with a greater frequency of snacking in New Zealand teenagers. It is possible that the cereal and milk intervention had the effect of reducing the consumption of fat-rich snacks; this is the subject of further investigation. Before the intervention, analysis indicated a significant interaction between various measures of carbohydrate intake; this seemed to be a result of between-group differences in mean values (not-significant) for these variables at baseline. The interaction was not evident post-intervention and we do not ascribe particular significance to the observation.

In conclusion, the daily consumption of a micronutrient-fortified breakfast cereal for 12 weeks, in the morning or the evening, by adolescent girls in the UK, improved status indices for vitamins B_2_, B_12_, folate, and iron. For each of these micronutrients this effect was significantly greater than benefits of consuming a non-fortified cereal. Vitamin D status was maintained in girls consuming fortified cereal in contrast with a decline seen in girls consuming unfortified cereal. Consuming cereal in the evening, whether fortified or non-fortified, elicited a modest weight gain but this was not associated with an increase in energy intake. To our knowledge this is the only randomised controlled trial of cereal intervention in adolescents reporting on dietary intake and micronutrient status. These findings are relevant across age and gender groups but have particular importance during the transition from childhood to adolescence, which is generally associated with less healthy dietary choices. The findings contribute to our understanding of the value of developing strategies to encourage the regular consumption of breakfast cereal in adolescents.

### Limitations of the study

Dietary intake was estimated using 4-day diet diaries, supported by food portion booklets; the use of weighed records would have produced more reliable data but the additional burden would probably have reduced ease of recruitment to the study and jeopardised compliance. In retrospect it would have been useful to have determined energy expenditure before and during the intervention, given the reported influence of breakfast consumption on morning energy expenditure. However, this would have entailed a significant demand on participants and may have reduced compliance. It may also have been useful to have explored the prevalence of mis-reporting of dietary data, although this is a complex subject to examine and the randomisation design should have minimised the impact on the main findings.

### Strengths of the study

To our knowledge this is the only randomised placebo controlled intervention trial that has attempted to determine the effects of consuming breakfast cereal and milk on micronutrient intake and status in adolescents. The separation of the intervention into a morning and an evening consumption added value in that this allowed us to demonstrate the potential benefits of consuming cereal with milk in an evening as an alternative to morning consumption.
